# Syndecans and Enzymes Involved in Heparan Sulfate Biosynthesis and Degradation Are Differentially Expressed During Human Odontogenesis

**DOI:** 10.3389/fphys.2018.00732

**Published:** 2018-06-14

**Authors:** Darko Kero, Tanja Simic Bilandzija, Lidija Lasic Arapovic, Katarina Vukojevic, Mirna Saraga-Babic

**Affiliations:** ^1^Department of Dental Morphology and Anthropology, Study Program of Dental Medicine, School of Medicine, University of Split, Split, Croatia; ^2^Department of Maxillofacial Surgery, University Clinical Hospital Mostar, Mostar, Bosnia and Herzegovina; ^3^Study Program of Dental Medicine, School of Medicine, University of Mostar, Mostar, Bosnia and Herzegovina; ^4^Primary Health Care Center Mostar, Mostar, Bosnia and Herzegovina; ^5^Laboratory for Early Human Development, Department of Anatomy, Histology and Embryology, School of Medicine, University of Split, Split, Croatia

**Keywords:** syndecans, heparan sulfate, extracellular matrix, human tooth germ, odontogenesis

## Abstract

Syndecans belong to a four-member family of cell surface heparan sulfate proteoglycans (HSPGs) abundantly present in various tissues. They are primarily recognized as extracellular matrix (ECM) receptors able to bind various ECM components and form gradients of morphogens and growth factors. Syndecans are composed of core protein with distinctive cytoplasmic, transmembrane, and extracellular domains to which several HS glycosaminoglycan (GAG) chains are covalently attached. In development of composite organs, such as teeth, expression patterns of syndecans display temporo-spatial shifts between epithelial and mesenchymal tissue compartments. Along with diverse functional properties of syndecans and generally large number of their interactors due to HS GAG chain content, this suggests possible involvement of syndecans in modulation of epithelial-to-mesenchymal crosstalk. Functional versatility of syndecans greatly depends upon the biochemical properties of attached HS GAG chains. These are specifically determined during the HS biosynthesis by the combinatorial action of glycosyl-transferases (Exts/EXTs) and bi-functional sulfotransferases (Ndsts/NDSTs), as well as by post-biosynthetic enzymatic cleavage of HS by the only active endoglucuronidase in mammals, heparanase 1 (Hpse1/HPSE1). Matching the essential requirement for HS during organogenesis, null-mutant animals for genes encoding these enzymes display severe developmental anomalies of mineralized tissues (including teeth) with embryonic or perinatal lethality. In this study, we analyzed expression of syndecan HSPGs (syndecans 1, 2, and 4), enzymes involved in HS biosynthesis (EXT1, NDST1, NDST2) and HS cleavage (HPSE1) in human tooth germs during the early stages of odontogenesis. All of the investigated factors displayed temporo-spatial differences in expression patterns, and some of them showed distinctive asymmetries of expression domains. Our findings suggest that these factors might be differentially involved in cellular processes which take place during the early odontogenic sequence in humans.

## Introduction

Syndecans belong to a small family of cell surface heparan sulfate proteoglycans (HSPGs) and are one of the most abundant cell surface receptors (Gotte, 2003; Stepp et al., 2015). Biochemically, syndecans are glycoconjugates composed of a core protein whose ectodomain is immersed in the extracellular matrix (ECM) and to which several HS glycosaminoglycan (GAG) side chains are covalently attached (Taylor and Gallo, 2006). Syndecans have multiple functions, but are primarily recognized as ECM receptors and growth factor co-receptors due to their ability to bind various ECM components and growth factors via their HS GAG side chains (Bernfield et al., 1992; Roper et al., 2012; Teng et al., 2012). In adult tissues, syndecans are differentially expressed in the epithelial linings of organs and body cavities (syndecan 1 – Sdc1), connective tissue (syndecan 2 – Sdc2), neural tissues (syndecan 3 – Sdc3), or have ubiquitous expression (syndecan 4 – Sdc4). However, in development of composite organs such as teeth, their expression can transiently shift between different tissue compartments in a temporo-spatial manner (Salmivirta et al., 1991; Thesleff and Mikkola, 2002; Thesleff, 2003). During the early stages of odontognesis, expression of Sdc1 shifts from epithelial to mesenchymal parts of tooth germ on a stage-specific basis. This is indicative of epithelial-to-mesenchymal crosstalk, through which the exchange of inductive odontogenic potential between the tooth germ tissue compartments occurs. The importance of epithelial-to-mesenchymal crosstalk for normal progression of tooth development has been demonstrated by numerous tissue recombination experiments, whereas its early disruption causes arrest of odontogenesis as observed in null-mutant animals for genes regulating jaw patterning and tooth number (Sharpe, 2001; Miletich and Sharpe, 2003; Chen et al., 2009; Ohazama et al., 2009; Kero and Saraga-Babic, 2016).

In developing tissues, syndecans are involved in regulation of cellular responses to challenges from microenvironment (ECM dynamics), which determine cell fate, cell shape, proliferative potential and the ability of cells to migrate. This functional versatility of syndecans is intricately associated with biochemical properties of their HS GAG side chains (Li and Vlodavsky, 2009). Biosynthesis of HS is a multistep process executed by a number of enzymes organized in physical complexes. Exostosin glycosyl-transferases (Exts/EXTs) and bi-functional deacetylase-N-sulfotransferases (Ndsts/NDSTs) have key roles in HS biosynthesis (Stickens et al., 2000; Holmborn et al., 2004). Exts/EXTs are needed for elongation of HS chain by adding repeating disaccharide units, whereas Ndsts/NDSTs modify HS by sulfation at specific residues. Sulfation patterns created by Ndsts/NDSTs are required for all subsequent modifications of HS including the post-biosynthetic glycosidic cleavage by endoglucuronidase heparanase 1 (Hpse1/HPSE1) (Poon et al., 2014). This, in turn, determines the HS-binding properties, and consequently the HSPGs ability to bind ECM components and form HS-binding factors gradients (Yasuda et al., 2010). Matching the essential requirements for HS during the early development, Exts/EXTs, Ndsts/NDSTs, and Hpse1/HPSE1 are ubiquitously expressed in all embryonic and fetal tissues, whereas some of these enzymes have non-redundant functions in normal development. Thus, the loss-of-function mutations of encoding genes are associated with early embryonic lethality (Ext1 knockout mice), or perinatal lethality (Ndst1 knockout mice, Ndst1/2 double knockouts) (Pallerla et al., 2007). The analyses of mutant animals disclosed that impaired biosynthesis of HS renders developing tissues unable to form proper gradients of morphogens and growth factors, so that co-ordination of signaling cues within the major signaling cascades (Wnt, Hh, Fgf, Bmp) gets severely disrupted.

Several studies have already indicated that Sdc1 is involved in modulation of multiple cellular processes during the odontogenesis in experimental animals and humans (Salmivirta et al., 1991; Filatova et al., 2014; Kero et al., 2014a, 2015). The aim of this study was to compare the expression patterns of Sdc1 with the expression patterns of other two family members, Sdc2 and Sdc4, during the early stages of human odontogenesis. We also sought to interpret these findings in a broader context by examining the expression patterns of factors involved in HS biosynthesis (EXT1, NDST1, NDST2) and degradation (HPSE1). These factors are functionally associated with syndecans, but the detailed description of their involvement in development of human tooth germ is still lacking. In general, HS GAG structure and content greatly determine the repertoire of syndecans’ interactors in the ECM, which may also be indicative of why syndecans might play versatile roles in molecular regulation of cellular processes in developing odontogenic tissues.

## Materials and Methods

### Tissue Procurement and Processing

For this study, we used fetal tissues from ten human conceptuses aged 7/8 (*n* = 3), 11/12 (*n* = 3), and 14 weeks of gestation (*n* = 4), containing tooth germs in the late bud, cap and early bell stages of development, respectively. Fetal tissue was obtained after spontaneous abortions and tubal pregnancies from the Department of Pathology, University Hospital Split, Split, Croatia. The samples were stored as histological sections at -24^o^C as a part of fetal human tissue archival collection of the Department of Anatomy, Histology and Embryology (University of Split, School of Medicine). Approval for tissue processing was given by the Ethical and Drug Committee of University Hospital Split (Class: 033-081/11-03/0005, No: 2181-198-03-04/10-11-0024, 2011) in accordance with Helsinki Declaration (Williams, 2008). Gestational age of human conceptuses was estimated by external measurements (O’Rahilly, 1972). Immunofluorescence staining was performed on tissues from craniocervical area or parts of jaw containing tooth germs. Paraffin-embedded tissue samples were cut in frontal or transversal planes (serial 7 μm sections) and mounted on glass slides for microscopic examination. Tissue preservation and presence of structures of interest was confirmed by examining control sections stained with haematoxylin and eosin (H&E staining). Following that, sections were processed for single and double immunofluorescence staining.

### Immunofluorescence Staining Protocol

Tissue sections were deparaffinized by standard protocol and further processed for single and double immunofluorescence as previously described (Kero et al., 2014b, 2016). Primary antibodies used for this study were: mouse monoclonal anti-Sdc1 [B-A38] (1:100, ab34164, Abcam, United Kingdom), rabbit polyclonal anti-Sdc2 (1:200, ab191062, Abcam, United Kingdom), rabbit polyclonal anti-Sdc4 (1:100, ab24511, Abcam, United Kingdom), rabbit polyclonal anti-EXT1 (1:100, ab126305, Abcam, United Kingdom), rabbit polyclonal anti-NDST1 (1:50, ab129248, Abcam, United Kingdom), rabbit polyclonal anti-NDST2 (1:100, ab151141, Abcam, United Kingdom), and rabbit polyclonal anti-HPSE1 (1:200, ab85543, Abcam, United Kingdom). Secondary antibodies were used at 1:400 dilution – anti-mouse Alexa Fluor 488 (GREEN, ab150105, Abcam, United Kingdom), anti-rabbit Alexa Fluor 594 (RED, ab150092, Abcam, United Kingdom), and anti-rabbit Alexa Fluor 488 (GREEN, ab150077, Abcam, United Kingdom). Following the incubation with secondary antibodies, sections were stained with 4′6′-diamidino-2-phenylindole (DAPI) to stain cell nuclei. For positive control, sections of healthy adult human gingiva were first stained with individual primary antibodies (single immunofluorescence) and later for double immunofluorescence with six different primary antibody tandems (all paired with anti-Sdc1). The same tandems of primary antibodies were subsequently used for double immunofluorescence staining of archival fetal tissue sections. Negative control staining was also performed by omitting the primary antibodies from the staining protocol. In total, 33 slides were used for immunofluorescence staining (27 – embryonic tissues; 4 – positive control; 2 – negative control) containing 111 tissue sections.

### Photo-Micrograph Acquisition and Initial Processing

Photo-micrographs were shot by SPOT Insight digital camera (Diagnostic Instruments, United States), mounted on Olympus BX61 fluorescence microscope (Olympus, Tokyo, Japan). Camera settings were set using image acquisition software CellA^®^ at 1360 × 1024 resolution, exposition of 1/1000 s with noise reduction filter. The blocking of background (auto-fluorescence) and equalization of signal was firstly performed on positive control images by triple dark point level adjustment using custom made presets in Adobe Photoshop^®^ CS6. After each step, pixel intensity values were measured (ImageJ, NIH Public Domain) at multiple marker points placed in structures known to be negative for staining with primary antibodies (cell nuclei), or DAPI (inter-nuclear spaces). Depending on the tissue type (epithelial/mesenchymal) and structure (cellular density, inter-nuclear space width), background reduction within 0–15 pixels was considered sufficient. Next, the fluorescence leakage reduction was performed by subtraction of counter-signals (red for green fluorescence and green for red fluorescence) with subsequent median signal intensity filtering in ImageJ.

### Co-localization and Intensity Distribution Analysis

Preparation of merged image doublets used for co-localization analysis, and determination of region of interest (ROI) was done as described previously (Kero et al., 2017). According to recommendations for fluorescence image analysis in biological microscopy, co-localization analysis was performed at magnification ×40 in order to eliminate co-localization/co-occurrence bias present at lower magnifications (Zinchuk and Zinchuk, 2008; Dunn et al., 2011). For intensity distribution analysis, thresholds were set for every photo-micrograph at seven different intensities – 15, 30, 45, 55, 70, 85, and 95 pixels. Threshold 8-bit images were then colorized in red (lower pixel value) and green (higher pixel value), merged and subtracted in order to exclude the overlapping (yellow) area. Thus, six new images were created displaying area covered by signals within specific intensity range in pixels, re-colorized to dark blue (15–30), bright blue (30–45), dark green (45–55), bright green (55–70), dark red (70–85), bright red (85–95), and yellow (95–255). These images were merged in order to create composite hotmaps of signal intensity distribution, and were also used to calculate the total size of individual factor expression domains (ImageJ, Adobe Photoshop^®^ CS6) (Supplementary Material). However, the additional sample unit threshold 8-bit images were made for each investigated factor within the same intensity range (15–95 pixels). The images were processed as described above, with exception that the subtractions of overlapping yellow areas were performed at five pixel increments (15–20, 20–25, 25–30, etc.). Thus, the total number of 204 sample unit threshold 8-bit images were made for each investigated factor (all observed stages of development included) and were subsequently used for statistical analysis of expression domain area distribution.

### Densitometry – 3D Surface and Plot Profiles

In order to create 3D surface plots and plot profiles, photo-micrographs were first calibrated to measurement scale in centimeters. Following that, the intensity in pixel values was converted to optical density (OD) units using Kodak No. 3 calibrated step tablet scanned with Epson Expression 1680 professional scanner. The tablet has 21 steps with a density range of 0.05–3.05 OD, however, total of 19 measurements is sufficient in order to obtain conversion curve with the list of corresponding intensity values in pixels and OD (ImageJ). Opposite from pixel value scale, increasing values in OD scale correspond to decreasing intensity. 3D surface plots were used to display the spatial distribution of signals represented by colored spikes corresponding to variations in intensity. Profile plots were used for the comparison of the overall intensity of expression, and to provide general information about the texture of staining patterns. Merged plot profiles with four intensity curves (corresponding to different stages of development and structures of interest) were made for each investigated factor.

### Statistical Analysis

Total surface covered by expression domain and the distribution of intensity (both expressed in percentage points) were calculated for individual factors, as well as for two groups of factors (syndecans/HSPGs and HS biosynthesis/degradation) in each ROI (whole tooth germs, cervical loop and inner enamel epithelium with surrounding mesenchymal tissues) (ImageJ). Thus, the comparative analyses were performed for three stages of development: late bud (7/8 weeks), cap (11/12 w), and early bell stage (14 w). Data distribution was analyzed by Kruskal–Wallis test, followed by Dunn’s Multiple Comparison Test (GraphPad Software, La Jolla, CA, United States). Statistical significance was set at *p* < 0.05.

## Results

**FIGURE 1:**
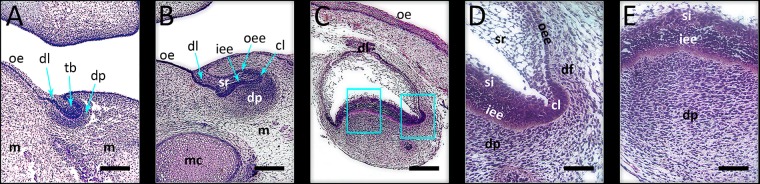
Investigated developmental stages of human incisor tooth germ. **(A)** late bud stage, 7/8 weeks of gestation; **(B)** cap stage, 11/12 weeks of gestation; **(C)** early bell stage, 14 weeks of gestation; magnified details of cervical loop **(D)** and inner enamel epithelium at the future cusp tip area **(E)** with surrounding tissues in human incisor tooth germ during the early bell stage (haematoxylin/eosin staining). During these stages, morphogenesis of enamel organ takes place, and is followed by histo-differentiation of enamel organ during the late bell stage. Note the significant increase of size of the enamel organ during the early bell stage compared to bud and cap stages of development. Magnifications: **(A–C)** × 10, *scale bar*: 240 μm; **(D,E)** × 40, *scale bar*: 60 μm. Designations: oe, oral epithelium; dl, dental lamina; tb, tooth bud; m, jaw mesenchyme; mc, Meckel’s cartilage; oee, outer enamel epithelium; iee, inner enamel epithelium; cl, cervical loop; sr, stellate reticulum; si, stratum intermedium; dp, dental papilla; df, dental follicle.

Tooth germ undergoes several histologically distinctive stages of development during the early odontogenic sequence (bud, cap, and bell) in which morphogenesis of enamel organ takes place. In this study, we analyzed expression domains and patterns of syndecans (Sdc1, Sdc2, and Sdc4) and enzymes involved in biosynthesis (EXT1, NDST1, and NDST2) and cleavage (HPSE1) of HS GAGs in human incisor tooth germs during the late bud (7/8 gestational weeks), cap (11/12 gestational weeks), and early bell stage (14 gestational weeks) of development (**Figure 1**).

### Expression of Sdc1, Sdc2, and Sdc4 in Human Incisor Tooth Germ During the Late Bud, Cap, and Early Bell Stages

**FIGURE 2:**
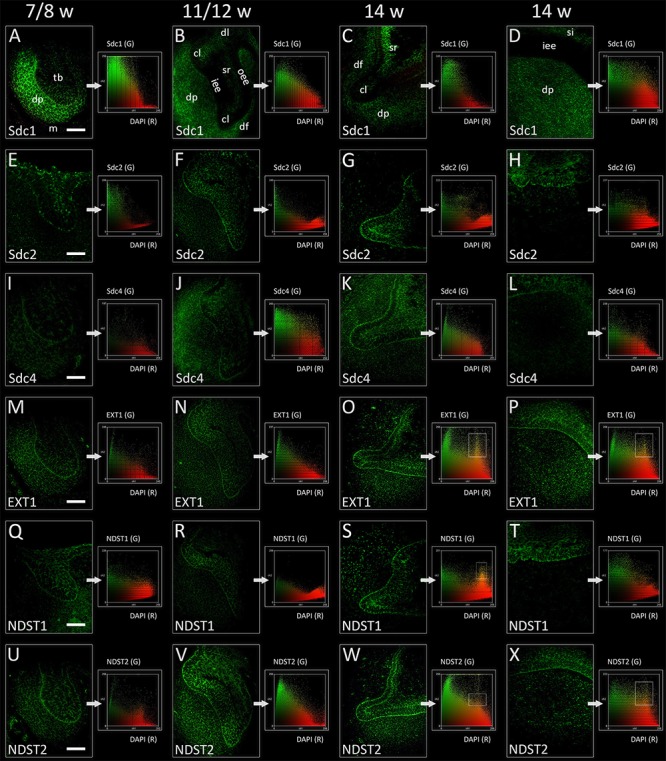
Expression of syndecans and HS biosynthesis enzymes in human incisor tooth germ during the late bud (7/8 w) **(A,E,I,M,Q,U)**, cap (11/12 w) **(B,F,J,N,R,V)**, and early bell stage (14 w) cervical loop **(C,G,K,O,S,W)** and inner enamel epithelium area **(D,H,L,P,T,X)** (immunofluorescence). Note that Sdc1, EXT1, and NDST2 expression domains display almost a mirror image compared with expression domain of NDST1 during the late bud stage **(A,M,Q,U)**. Arrows point to color scatterplots for co-localization of factor (GREEN channel) with 4′6′-diamidino-2-phenylindole (DAPI) nuclear stain (RED channel) for characterization of expression patterns (nuclear, non-nuclear/cytoplasmic/intercellular). Framed areas on color scatterplots for EXT1 **(O,P)**, NDST1 **(S)**, and NDST2 **(W,X)** point to co-localization (YELLOW, nuclear expression pattern) and thus nuclear expression pattern of these factors in cervical loops and inner enamel epithelium. Examination on maximum magnification showed that this is due to high density of cells in the tissues, and that EXT1, NDST1, and NDST2 in fact display perinuclear expression pattern. Magnification: **(A–X)** × 40, *scale bar*: 60 μm. Designations: oe, oral epithelium; dl, dental lamina; tb, tooth bud; m, jaw mesenchyme; oee, outer enamel epithelium; iee, inner enamel epithelium; cl, cervical loop; sr, stellate reticulum; si, stratum intermedium; dp, dental papilla; df, dental follicle.

**FIGURE 3:**
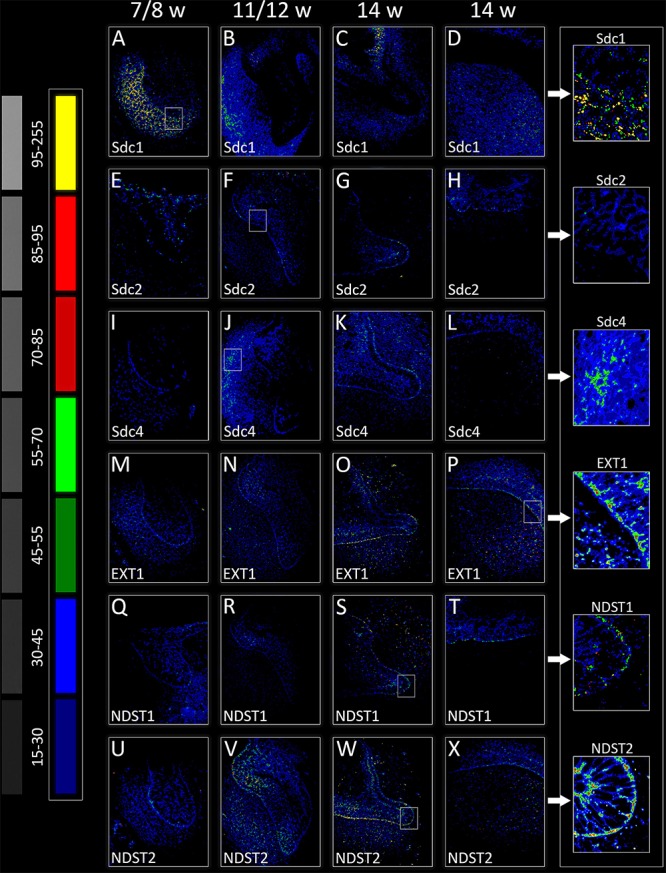
Intensity distribution hotmaps for syndecans and HS biosynthesis enzymes in human incisor tooth germ during the late bud (7/8 w) **(A,E,I,M,Q,U)**, cap (11/12 w) **(B,F,J,N,R,V)**, and early bell stage (14 w) cervical loop **(C,G,K,O,S,W)** and inner enamel epithelium area **(D,H,L,P,T,X)**. Far Left: colorized pixel intensity scale paired with seven corresponding mean values from Kodak No. 3 step calibration tablet. Arrows point to magnified framed areas on hotmaps for Sdc1 **(A)**, Sdc2 **(F)**, Sdc4 **(J)**, EXT1 **(P)**, NDST1 **(S)**, and NDST2 **(W)** at different stages of tooth development. Magnification: **(A–X)** × 40, *scale bar*: 60 μm. Intensity range (pixel value): 15–30 (dark blue), 30–45 (bright blue), 45–55 (dark green), 55–70 (bright green), 70–85 (dark red), 85–95 (bright red), and 95–255 (yellow).

In the late bud and cap stages, Sdc1 was transiently expressed in the mesenchymal parts of tooth germ (condensing mesenchyme, dental papilla, and dental follicle). Expression of Sdc1 in the epithelial parts of human tooth germs was almost absent during the bud stage, but was visible in the differentiating stellate reticulum during the cap stage, especially in its distal/posterior portion (**Figures 2A,B, 3A,B**). Similar pattern was observed in the early bell stage, albeit with slightly reduced intensity of staining in dental papilla and dental follicle surrounding the cervical loops. Stellate reticulum, as well the newly differentiated stratum intermedium (several condensed layers of spindle-shaped cells capping the inner enamel epithelium), were positive to Sdc1 (**Figures 2C,D, 3C,D**). Inner enamel epithelium, outer enamel epithelium, and cervical loops were negative to Sdc1 staining during the cap and early bell stage. Expression domains of Sdc2 and Sdc4 displayed only partial overlapping with expression domain of Sdc1. However, unlike Sdc1, both factors were expressed on the epithelial side of epithelial-mesenchymal interface (outer rim of the tooth bud, inner and outer enamel epithelia of enamel organ) with variable intensity throughout the entire investigated period (**Figures 2E–L, 3E–L**). In contrast to Sdc2, Sdc4 was expressed in parts of dental follicle adjacent to cervical loops, and expression of both Sdc and Sdc4 was visible throughout the inner enamel epithelium and stratum intermedium in the early bell stage. Intensity correlation analysis, performed on single and double immunofluorescence images (Sdc1/Sdc2; Sdc1/Sdc4), disclosed non-nuclear expression pattern for Sdc1, Sdc2, and Sdc4 (co-localization with DAPI) (**Figure 2** CSP columns). Also, Sdc1 did not co-localize with Sdc2 throughout the investigated period, whereas the co-localization of Sdc1 and Sdc4 was observed only in the dental papilla during the cap stage (data not shown).

### Expression of EXT1, NDST1, and NDST2 in Human Incisor Tooth Germ During the Late Bud, Cap, and Early Bell Stages

Expression domains of EXT1 and NDST2 almost completely overlapped during the entire investigated period, and were ubiquitously visible in epithelial and mesenchymal parts of developing human tooth germ (**Figures 2M–P,U–X, 3M–P,U–X**). The expressions of EXT1 and NDST2 were of comparable intensity, albeit the expression of NDST2 was generally higher during the bud and cap stage. Furthermore, both factors were strongly expressed at the epithelial-mesenchymal interface, in the inner enamel epithelium (stronger EXT1) and cervical loops (stronger NDST2) (**Figures 3O,P,W,X**). Surprisingly, expression domain of NDST1 was extremely limited throughout the investigated period (**Figures 2Q–T, 3Q–T**). During the bud stage, the expression domain of NDST1 slightly overlapped with the expression domains of EXT1 and NDST2. It was located in a portion of the tooth bud and in the restricted area of condensing dental mesenchyme underlying the tip of the tooth bud (**Figures 2M, 3M**). In fact, the strongest expression of NDST1 was observed in jaw mesenchyme remotely surrounding the tooth germ, as well as in Meckel’s cartilage (mc) during that stage of development (data not shown). In cap and early bell stages, NDST1 was mostly expressed in the inner epithelium and cervical loops. However, the intensity of expression was weaker than that of EXT1 and NDST2. Intensity correlation analysis disclosed non-nuclear expression pattern (co-localization with DAPI), except for a slight co-localization with DAPI in cervical loops (EXT1 and NDST2) and inner enamel epithelium (EXT1, NDST1, and NDST2) (**Figure 2** CSP columns) in the early bell stage. This was attributed to the extreme cell nuclei density of the inner enamel epithelium. Intensity correlation analysis performed at maximum magnification (×100) confirmed cytoplasmic expression pattern of all three factors (data not shown). As expected, color scatterplots for double immunofluorescence with Sdc1 (Sdc1/EXT1; Sdc1/NDST1; Sdc1/NDST2) disclosed no co-localization, and thus reconfirmed the cytoplasmic expression pattern of EXT1, NDST1, and NDST2, since HS biosynthesis enzymes normally reside within the ER and Golgi apparatus (McCormick et al., 1998; Presto et al., 2008).

### Co-localization of Sdc1 and HPSE1 in Human Incisor Tooth Germ During the Late Bud, Cap, and Early Bell Stages

**FIGURE 4:**
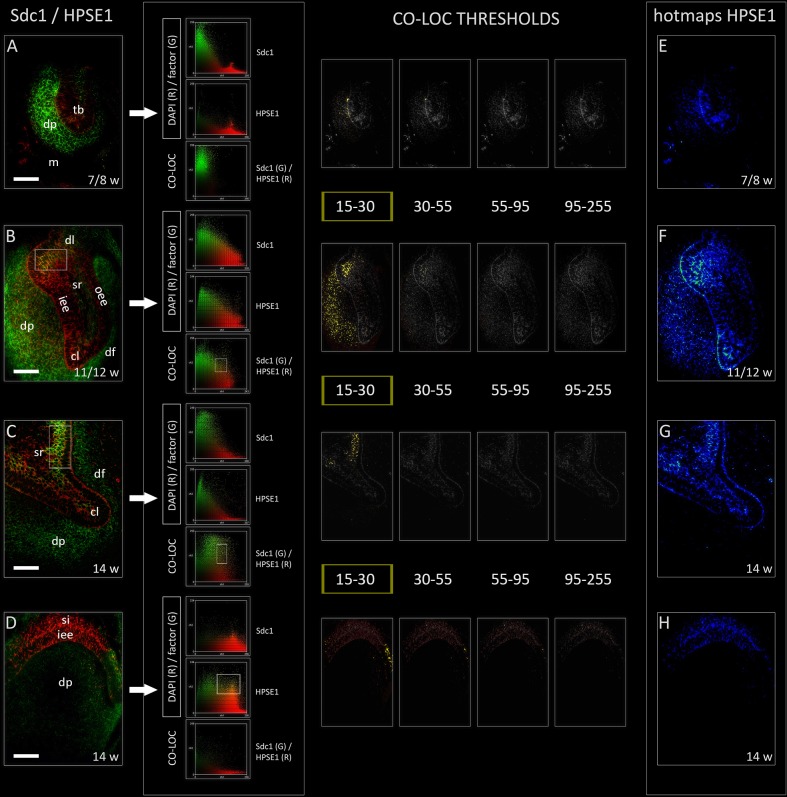
Expression of HPSE1 and double immunofluorescence Sdc1/HPSE1 in human incisor tooth germ during the late bud (7/8 w), cap (11/12 w) and early bell stage (14 w) cervical loop **(C)** and inner enamel epithelium area **(D)**. Arrows point to color scatterplots for co-localization of factor (GREEN channel) with DAPI nuclear stain (RED channel), and co-localization of Sdc1 (GREEN channel) with HPSE1 (RED channel) for all investigated stages. HPSE1 displays cytoplasmic expression pattern during the bud stage **(A)**, whereas in cap and early bell stages it is expressed in inter-nuclear space (ECM) (co-localization with Sdc1) **(B–D)**. Co-localization with Sdc1 occurs in baseline intensity range (15–30 pixels) (YELLOW color) (CO-LOC THRESHOLDS section), and in the cap stage displays asymmetric pattern in the epithelial part of tooth germ (posterior/distal portion). Note that the expression domains of Sdc1 and HPSE1 are well demarcated during the bud stage **(A)**, and in the inner enamel epithelia area **(D)** during the early bell stage. Far right column: intensity distribution hotmaps for HPSE1 in human incisor tooth germ during the bud, cap, and early bell stage of development **(E–H)**. Magnification: **(A–X)** × 40, *scale bar*: 60 μm. Intensity range (pixel value): 15–30 (dark blue), 30–45 (bright blue), 45–55 (dark green), 55–70 (bright green), 70–85 (dark red), 85–95 (bright red), and 95–255 (yellow).

Heparanase 1 was mostly expressed in the epithelial parts of human tooth germ, apart from its ubiquitous expression during the cap stage (**Figures 4A–H**). The most intense staining was observed in the cervical loops during the cap stage, and in small sections of stellate reticulum adjacent to the cervical loops and outer enamel epithelium (**Figures 4F,G**). In the early bell stage, expression of HPSE1 in the cervical loops was less intense and limited to their tips. Furthermore, the expression of HPSE1 in the area of future cusp tip was visible in the inner enamel epithelium and overlaying stratum intermedium. In this area, the expression of HPSE1 was clearly demarcated on one side, whereas on the other side it displayed gradual decrease in intensity toward the cervical loop (**Figure 4D**). However, throughout the entire investigated period, epithelial aspect of the epithelial-mesenchymal interface between the enamel organ and underlying dental papilla was positive to HPSE1. Intensity correlation analysis disclosed non-nuclear expression pattern (co-localization with DAPI), except for a slight co-localization in cervical loops and the inner enamel epithelium in the early bell stage. No co-localization with DAPI was found when the intensity correlation analysis was performed on maximum magnification (×100). In contrast to EXT1, NDST1, and NDST2, color scatterplots for double immunofluorescence with Sdc1 disclosed co-localization during the cap stage, and in cervical loops during the early bell stage (**Figure 4** CSP column). Taken together, this means that HPSE1 displays both cytoplasmic and intercellular expression patterns in developing human tooth germ. Serial mergers of four HPSE1 photo-micrographs (thresholds set from low to high signal intensity) with Sdc1 photomicrograph (threshold set at baseline intensity value) disclosed the co-localization patterns at low intensity of HPSE1 expression. Co-localization pattern was particularly visible during the cap stage in a wide area of dental papilla arching toward the distal/posterior part of the enamel organ. Thus, co-localization of Sdc1 and HPSE1 expression domains displayed somewhat asymmetric distribution (**Figure 4** CO-LOC THRESHOLDS).

### Densitometry Profiling and Statistical Analysis of Expression Domains

**FIGURE 5:**
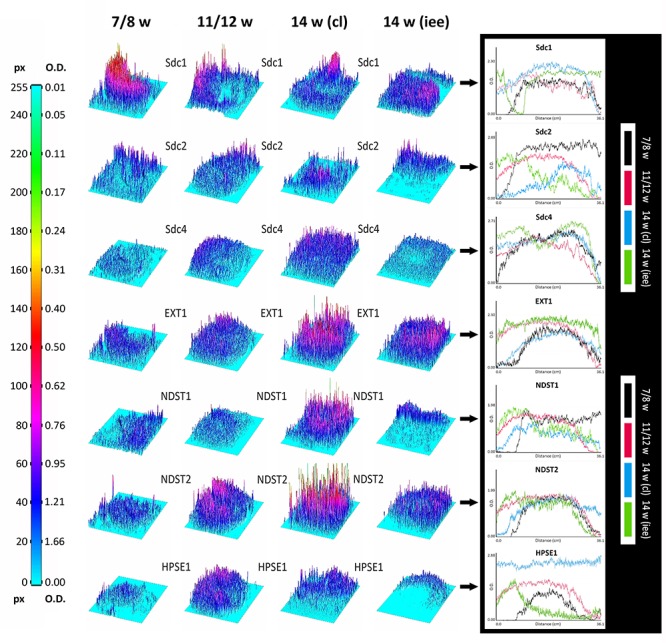
3D surface plots and intensity plot profiles for all investigated factors in human incisor tooth germs during the late bud (7/8 w), cap (11/12 w) and early bell stage (14 w) cervical loop and inner enamel epithelium area. Far Left: colorized full-range intensity scale with pixel values and corresponding values in OD units. Arrows point to merged intensity plot profiles of scanned photo-micrographs (image size calibrated in cm) for each investigated factor. Distance/cm (*x*-axis) represents image height (scanned vertically), intensity in OD (*y*-axis). Increase in OD value corresponds to decrease in intensity (opposite from pixel value). Intensity plot profiles are informative of staining patterns. Well-limited expression patterns (nuclear, perinuclear, non-diffuse cytoplasmic) display curves with closely aligned spikes (such as EXT1, NDST1, NDST2, and HPSE1 known to be located in ER, Golgi or lysosomes), whereas flat staining (diffuse cytoplasmic, membranar and inter-nuclear expression patterns) produces stretched curves (see Sdc1, Sdc2, and Sdc4 plot profiles).

Surface plots and vertical plot profiles disclosed stage-specific variations in expression distribution and intensity for all investigated factors (**Figure 5**). These may be attributable to the involvement of investigated factors in highly dynamic cellular processes during the progression of morphogenesis of enamel organ. Among syndecans, the expression of Sdc1 continuously displayed the greatest range in both the intensity and spatial distribution. In contrast to Sdc2, the intensity of Sdc1 expression slightly decreased in intensity as the tooth germ development progressed and odontogenic tissues began to differentiate. Sdc4 displayed relatively balanced intensity profile. Intensity profiles of EXT1, NDST1, and NDST2 disclosed that the HS biosynthesis is differentially regulated in a stage-specific manner. While the expression of EXT1 and NDST2 displayed well-matched profiles, that of NDST1 indicated more fluctuations in intensity and limited spatial distribution. HS biosynthesis enzymes operate in physical complexes, whose composition greatly determines the structure of HS (Esko and Selleck, 2002). This means that the biochemical properties of HS, as well as the properties of HSPGs such as syndecans, might significantly vary between different tissue compartments of developing human tooth germ.

**FIGURE 6:**
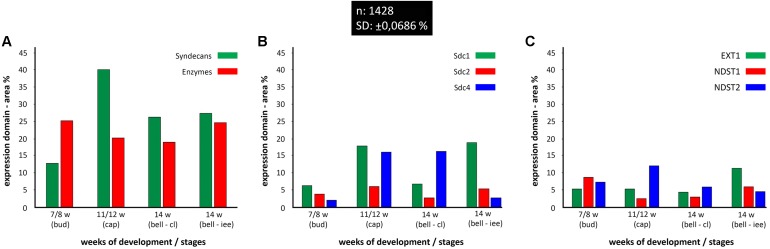
Size expression domains of all investigated factors in human incisor tooth germ during the late bud (7/8 w), cap (11/12 w), and early bell stage (14 w) cervical loop (cl) and inner enamel epithelium area (iee). Comparison was made for stages of development and structures of interest (*x*-axis). Expression domain size was calculated as a fraction of total ROI area and expressed in percentage (%) (*y*-axis; SD: ± 0,0686%) from sample unit threshold 8-bit images. The total of 1428 sample unit threshold 8-bit images (*n*) were used. Comparison of total expression domain size between syndecans and HS biosynthesis and degradation enzymes **(A)**; comparison of expression domain size between individual syndecans **(B)**; comparison of expression domain size between individual HS biosynthesis enzymes **(C)**.

Quantitative analysis of expression domain and intensity distribution disclosed statistically significant differences only for Sdc4 between late bud and early bell stage (cervical loop), as well as in the early bell stage between the cervical loop and inner enamel epithelium (*p* < 0.001, Kruskal–Wallis test). By comparison of these data with relatively balanced intensity of Sdc4 profile displayed throughout the investigated period, the upregulation of Sdc4 in cervical loop detected by quantitative analysis seems to be mostly due to the increased size of Sdc4 expression domain, and much less due to variation in intensity range of its expression (**Figures 5, 6**).

## Discussion

### HPSE1 Is a Putative Modulator of Syndecan Binding to ECM in Human Tooth Germ

The expression of Sdc1 in developing human tooth germ displays transient decrease in proliferating cervical loops from cap to early bell stage, but it is increased in the underlying mesenchyme of dental papilla and follicle. Thus, cervical loops are rendered loose enough to invade the mesenchyme, whereas the mesenchyme provides scaffolding for their directional ingrowth. However, this explanation falls short when trying to interpret the co-expression of Sdc2 and Sdc4 in cervical loops visible in human tooth germs during the early bell stage. Syndecans’ core protein ectodomains and HS GAG side chains are prone to shedding or glycosidic cleavage, respectively. This can promote the loosening of cellular attachment to ECM since the binding of syndecans to the ECM components goes via HS side chains. Furthermore, the cleavage of HS side chains may in turn facilitate shedding of the core protein ectodomain by ECM-resident proteinases such as MMPs (Bonnans et al., 2014). Interestingly, the expression of various MMPs in developing mouse tooth germs is reported to be mostly present at the epithelial-mesenchymal interface during the entire progression of enamel organ morphogenesis (Yoshiba et al., 2003).

For two reasons, it is difficult to say to what extent the expression patterns of syndecans described in this study can be attributed to the intact or cleaved syndecan molecules. Firstly, the antibodies against Sdc1, Sdc2, and Sdc4 target only their core proteins, and since their core protein ectodomains are disproportionally longer than the short transmembrane and cytoplasmic domains, staining produced by either of the antibodies uniformly labels cell membrane and surrounding ECM (Kero et al., 2014a). Secondly, the shedding of ectodomain usually takes place within a short amino-acid sequence located in close proximity to cell membrane, so conventional microscopy lacks sufficient resolution to discern between the labeling of intact and shed core protein. Also, we cannot discount the slight overlapping of staining due to extremely high cellular density in certain regions of human tooth germ (enamel epithelia, outer portions of condensing mesenchyme in dental papilla). However, the co-localization analysis of expression patterns of Sdc1 and HPSE1, as well as the comparison of expression domains of Sdc2, Sdc4, and HPSE1, do imply that the cleavage of HS side chains might occur during the early stages of human tooth germ development. Namely, Sdc1 and HPSE1 co-localize at low intensity range of HPSE1 expression, meaning that only a certain fraction of HPSE1 resides within the ECM of odontogenic tissues. This might be attributed to the observation that the activity of all ECM degrading enzymes must be tightly regulated in order to maintain balance in ECM dynamics (Lu et al., 2011). Interestingly, the co-localization pattern of Sdc1 and HPSE1 was asymmetric covering the posterior/distal area of developing human tooth germ. Previous reports on the expression of Sdc1 and 10E4 antibody (which binds specific motif found on intact HS side chains) in developing mouse tooth germs disclosed that their expression domains display almost a mirror image (Bai et al., 1994). However, the 10E4 antibody staining was abolished when performed on tooth germ tissue sections which were previously treated with heparitinase, the enzyme which cleaves the HS side chains to HS stubs. The commercial antibodies against HPSE1 cannot distinguish between the active and inactive forms of enzyme, and therefore, our findings only confirm that in developing human tooth germs, certain amounts of HPSE1 can be found in the ECM of tissues where Sdc1 is expressed. Furthermore, the presence or absence of enzymatic activity of HPSE1 cannot be simply confirmed based on the tissue compartment in which the HPSE1 staining is located. Apart from the extracellular spaces, both active and inactive forms of HPSE1 can also be found in cytoplasm where the enzyme accumulates in lysosomes (Gutter-Kapon et al., 2016).

### EXT1, NDST1, and NDST2 Differentially Regulate HS Biosynthesis in Human Tooth Germ

The ability of syndecans to modulate the gradients of morphogens and HS-binding growth factors partly depends on activity of enzymes such as HPSE1. By cleaving the HS side chains, HPSE1 can reduce the binding capacity of HSPGs and/or facilitate the release of HS-binding factors from the ECM storage (Goldberg et al., 2013; Stoler-Barak et al., 2014). However, there are two additional mechanisms through which HSPGs participate in establishing the gradients of morphogens and HS-binding factors in developing tissues – by formation of channels for directed diffusion of morphogens, or by acting as co-receptors which modulate the affinity of growth factors to their cognate receptors (Hynes, 2009; Rozario and DeSimone, 2010; Goetz and Mohammadi, 2013). The former mechanism stems from the physical properties of HS. Namely, HS is a highly charged molecule able to draw water, which causes the swelling of ECM and formation of diffusion channels. In turn, these channels direct the spreading of morphogens through ECM to target tissues. The importance of this mechanism is nicely demonstrated on knockouts for HS biosynthesis enzymes. For example, poorly directed (or inhibited) diffusion of Ihh morphogen due to impaired synthesis of HS in Ndst1 knockout mice is responsible for defective development of craniofacial skeleton affecting the temporomandibular joint, mandible and teeth (Pallerla et al., 2007). The latter mechanism of gradient formation, on the other hand, is based on biochemical properties of HS, i.e., its highly variable sulfation patterns, and is responsible for formation of more narrow and focused gradients, which is crucial for fine-tuning of epithelial-to-mesenchymal cross talk during morphogenesis. For example, in developing submandibular gland, certain members of FGF growth factor family bind with high affinity to HSPGs and, thus, are able to form narrow, focused gradients having only a portion of epithelium exposed to their action. However, the other FGFs which bind to HSPGs with low affinity, form broader gradients resulting with the formation of epithelial buds in multiple directions (Yasuda et al., 2010). Furthermore, the studies on effects of Ndst1 knockout in developing mouse eye have demonstrated that reduced ability of HSPG to act as FGFs co-receptors (due to insufficient sulfation of HS) severely disrupts binding of FGFs to their cognate receptors, and compromises multiple FGF signaling pathways resulting in defective eye development (Pan et al., 2006).

The structure of HS greatly varies between different cell types and tissues, but how that structure is determined is not clear. It seems that there is no template for HS biosynthesis, but according to the GAGosome concept, HS biosynthesis is primarily regulated through the combinatorial action of individual members of physical complexes formed by a subset of enzymes residing in the ER or Golgi apparatus (Esko and Selleck, 2002). Heart and kidney cell culture studies have shown that cells which constitutively overexpress Ext1, Ext2, or Ndst1 can synthesize HS with striking variety in degree and patterns of sulfation, and thus with very different physical and biochemical properties. Surprisingly, this cannot be simply correlated with the relative concentrations of these enzymes within their complexes. For example, the lowest degree of HS side chain sulfation was observed in cells overexpressing all three enzymes (Presto et al., 2008).

As expected, the expression patterns of EXT1, NDST1, and NDST2 in developing human tooth germs described in this study might suggest that the HS biosynthesis is active process during the early stages of human odontogenesis. The analyses of area size covered by these factors’ expression domains, as well as the intensities of their expression, also suggest that the differential enzymatic activity of EXT1, NDST1, and NDST2 could be patterned in a stage- and tissue-specific manner, which, in a broader context, might be related to the GAGosome concept. Even though Ndst1, unlike Ndst2 and Ndst3, is considered to be non-redundant for normal development, the expression domain of NDST1 in human tooth germ was surprisingly limited in comparison with those of EXT1 and NDST2. Furthermore, expression domain of NDST1 transiently displayed a mirror image asymmetry observed for expression domain of Sdc1, and partial overlapping with expression domains of Sdc2 and Sdc4. Related to data from the aforementioned studies, these findings might imply that the composition of HS could vary between different odontogenic tissues and stages of human tooth germ development. Furthermore, multiple and mutually distinctive roles attributed to individual syndecans in regulation of cellular processes during the odontogenesis might be derived from variations in composition and biochemical properties of their HS side chains. Since we worked with archive tissue sections, we were unable to apply protein blotting methods, which might disclose the possible physical interactions of HS biosynthesis enzymes, and thus better show the actual composition of their complexes in human odontogenic tissues. Additional research of expression patterns of other enzymes involved in HS biosynthesis and various antibodies against HS GAG chains is also necessary in this matter.

### Redundancy of Syndecans and Enzymes Involved in HS Biosynthesis and Degradation During Organogenesis

Differential expression of syndecans and enzymes involved in HS biosynthesis and degradation during human odontogenesis must be viewed within the context of a vast regulatory network whose operating and redundant mechanisms are far from being understood (Szatmari et al., 2012; Szatmari and Dobra, 2013; Afratis et al., 2017). Although individual syndecans display distinctive stage- and tissue-specific expression domains during organogenesis, single knockout mice for *Sdc1* or *Sdc4* develop normally, are fertile, have normal life span, and only fail in response to different challenges during adulthood (wound healing, inflammatory response) (Alexopoulou et al., 2007). In contrast, *Sdc2* single knockout zebrafish and *Xenopus laevis* display impairment of left-right asymmetry, but whether the ablation of *Sdc2* in mammals can produce similar phenotypes is not known, since, no *Sdc2* single knockout mice have been created and studied to date (Stepp et al., 2015). No discernible phenotype could also be seen in heparanase 1 (*Hpse1*) knockout mice even though Hpse1/HPSE1 is the only known active endo-β-glucuronidase in mammals found to be ubiquitously expressed during development (Zcharia et al., 2009). The absence of HPSE1 activity is supposed to have profound effects on cell behavior and tissue homeostasis, but the proposed compensatory mechanisms significantly differ based on genetic backgrounds of *Hpse1*/knockout mice (Poon et al., 2014). Loss-of-function mutations of genes encoding enzymes for HS biosynthesis, such as Exts and Ndsts, cause developmental anomalies with early lethality in mice (Yasuda et al., 2010). It is intriguing, however, that single *Ext1* knockout mice fail to gastrulate, whereas the loss-of-function mutations of human homolog *EXT1* show incomparably milder phenotype (multiple hereditary exostosis). Therefore, while these enzymes perform similar functions in mice and humans due to their homology, the redundant mechanisms covering for their loss-of-function might significantly differ between the two species.

Due to ethical considerations and scarcity of specimens, research of early human development is by default methodologically limited and descriptive. Therefore, based on results presented in this study, it is not possible to fully assess the functional importance of investigated factors for normal development of human tooth germs. Most of what we know about molecular regulation of odontogenesis comes from studies of developing mouse molars. However, inter-species differences in dental formula, duration of odontogenic sequence and morphology of fully developed teeth, do suggest that more or less subtle differences in molecular regulation of odontogenesis in different species should be reflected on molecular level. For that matter, the present and future studies of involvement of syndecans and functionally related factors in cellular processes during human tooth development are not without the intrinsic value, because they might also provide some clues into how these differences are established.

## Author Contributions

DK designed the study, devised and selected image analysis methods, performed selection of primary antibodies and control immunofluorescence staining, processed and interpreted data, reviewed the literature, and wrote the manuscript. TSB performed and supervised immunofluorescence staining, acquired and interpreted data (immunofluorescence, plot profiles), wrote parts of the manuscript (sections “Materials and Methods” and “Results”), reviewed part of the literature, and proof-read the manuscript. LLA performed immunofluorescence staining, assisted in acquisition of data and initial photo-micrograph processing, reviewed part of the literature, and proof-read the manuscript. KV supervised immunofluorescence staining, reviewed part of the literature, interpreted data (statistical analysis), and proof-read the manuscript. MS-B provided theoretical background for the study design and revised and co-edited the manuscript with DK.

## Conflict of Interest Statement

The authors declare that the research was conducted in the absence of any commercial or financial relationships that could be construed as a potential conflict of interest.

## Supplementary Material

FIGURE S1 The making of intensity distribution 6-color hotmap. Initially processed photo-micrograph **(A)** is converted to 8-bit image **(B)**. 8-bit image is further adjusted at different intensity thresholds (SIGNAL THRESHOLDS section). For the purpose of this presentation, bright levels on 8-bit image were slightly increased in order to get better view of the intensity range at higher pixel values (this was not done with photo-micrographs analyzed in this study). Threshold pairs are chosen by visible differences in area coverage as follows: 15, 30, 45, 55, 70, 85, and 95 pixels. The subtraction of signal by customized preset in Adobe Photoshop^®^ CS6 (INTENSITY RANGE AREA SUBTRACTION section) goes in following threshold pairs: 15–30, 30–45, 45–55, 55–70, 70–85, and 85–95. These are colorized to dark blue (15–30), bright blue (30–45), dark green (45–55), bright green (55–70), dark red (70–85), and bright red (85–95). Colorized subtraction images are then sequentially merged from dark blue to bright red making the 6-color hotmap (HOTMAP Syndecan 1). Thus, we are able to get a better view of intensity distribution of particular immunofluorescence staining.

FIGURE S2 Expression pattern profiling of syndecans, enzymes involved in HS biosynthesis and HPSE1 in developing tooth germs during the investigated stages. (SECTION 1) Co-localization with DAPI nuclear staining; (SECTION 2) Intensity correlation analysis of expression patterns and the making of color scatter-plot **(A–F)**, NDST2 staining in developing human tooth germ during the cap stage (11/12 w), magnification ×40; (SECTION 3) Signal overlap analysis for NDST2 **(B,C)** and EXT1 **(E,F)** staining in the future cusp tip area of inner enamel epithelium (iee) of developing human tooth germ during the early bell stage (14 w); DAPI staining **(A,D)** reveals high nuclear density. Both NDST2 and EXT1 display cytoplasmic expression patterns. The reduction of signal overlap corresponds with higher magnification – compare color scatter-plots for NDST2/DAPI and EXT1/DAPI color scatter-plots [magnification **(A–C)**: ×100; details on **D–F** are additionally magnified]. Thick arrows (white, black with white stroke) point to the photo-micrograph processing sequence; thin arrows point to the tissue/structure of interest.
