# Supplementary figures and images for: Syndecans and Enzymes Involved in Heparan Sulfate Biosynthesis and Degradation Are Differentially Expressed During Human Odontogenesis
Source: Front Physiol. 2018 Jun 14;9:732. doi: 10.3389/fphys.2018.00732 (PMC6010574; doi:10.3389/fphys.2018.00732)

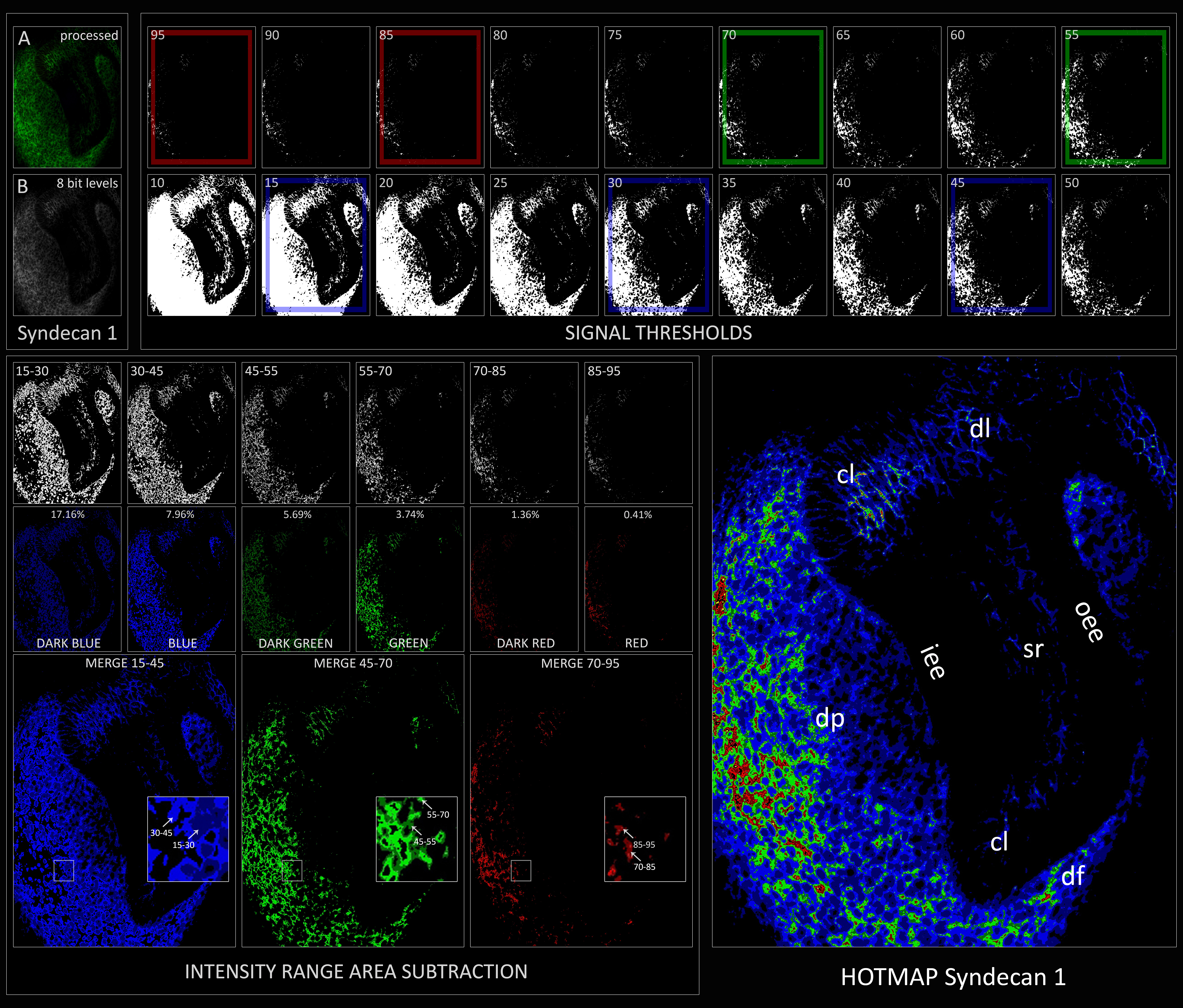

Supplement: FIGURE S1 — The making of intensity distribution 6-color hotmap. Initially processed photo-micrograph (A) is converted to 8-bit image (B). 8-bit image is further adjusted at different intensity thresholds (SIGNAL THRESHOLDS section). For the purpose of this presentation, bright levels on 8-bit image were slightly increased in order to get better view of the intensity range at higher pixel values (this was not done with photo-micrographs analyzed in this study). Threshold pairs are chosen by visible differences in area coverage as follows: 15, 30, 45, 55, 70, 85, and 95 pixels. The subtraction of signal by customized preset in Adobe Photoshop® CS6 (INTENSITY RANGE AREA SUBTRACTION section) goes in following threshold pairs: 15–30, 30–45, 45–55, 55–70, 70–85, and 85–95. These are colorized to dark blue (15–30), bright blue (30–45), dark green (45–55), bright green (55–70), dark red (70–85), and bright red (85–95). Colorized subtraction images are then sequentially merged from dark blue to bright red making the 6-color hotmap (HOTMAP Syndecan 1). Thus, we are able to get a better view of intensity distribution of particular immunofluorescence staining. [file Image_1.JPEG]

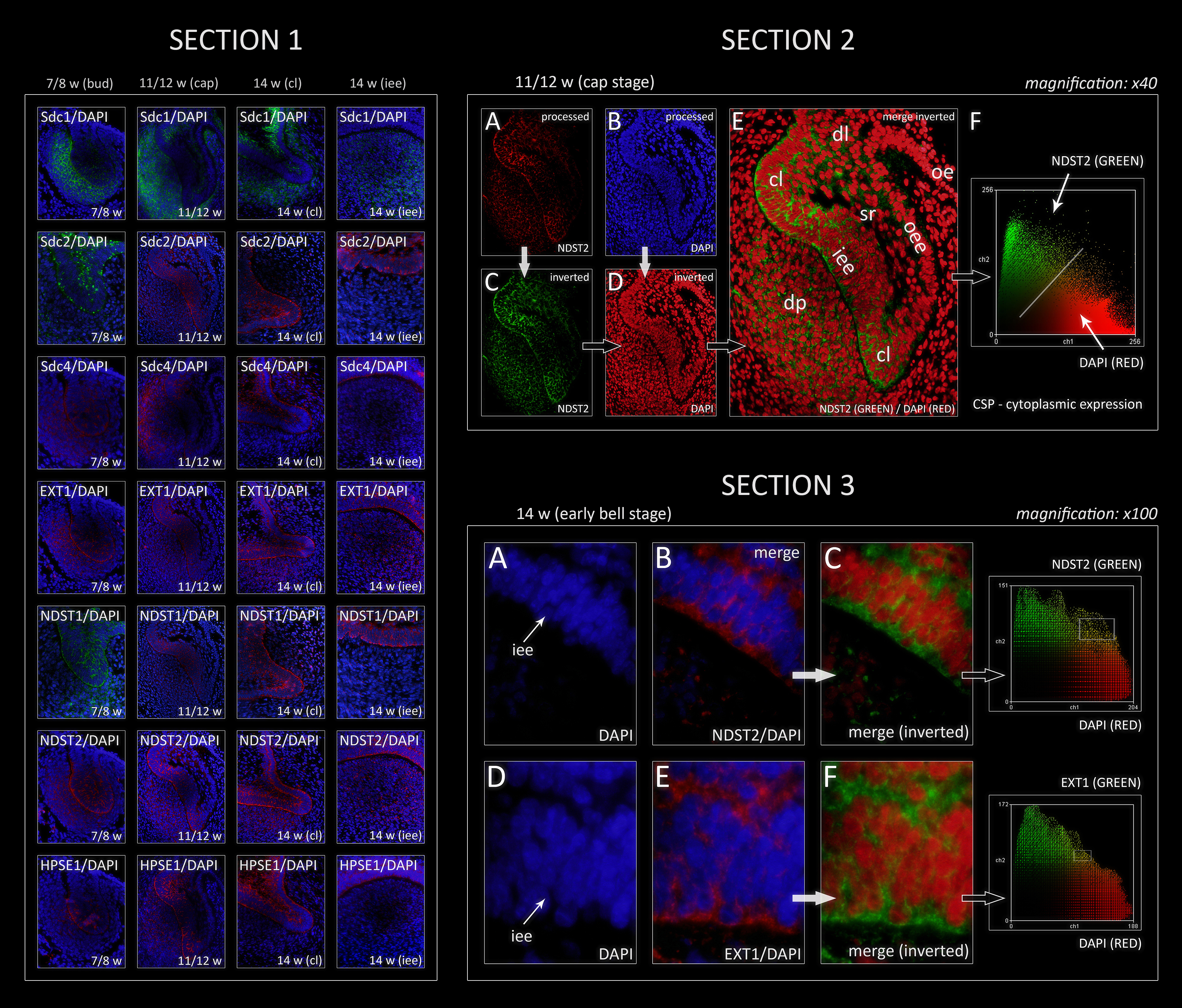

Supplement: FIGURE S2 — Expression pattern profiling of syndecans, enzymes involved in HS biosynthesis and HPSE1 in developing tooth germs during the investigated stages. (SECTION 1) Co-localization with DAPI nuclear staining; (SECTION 2) Intensity correlation analysis of expression patterns and the making of color scatter-plot (A–F), NDST2 staining in developing human tooth germ during the cap stage (11/12 w), magnification ×40; (SECTION 3) Signal overlap analysis for NDST2 (B,C) and EXT1 (E,F) staining in the future cusp tip area of inner enamel epithelium (iee) of developing human tooth germ during the early bell stage (14 w); DAPI staining (A,D) reveals high nuclear density. Both NDST2 and EXT1 display cytoplasmic expression patterns. The reduction of signal overlap corresponds with higher magnification – compare color scatter-plots for NDST2/DAPI and EXT1/DAPI color scatter-plots [magnification (A–C): ×100; details on D–F are additionally magnified]. Thick arrows (white, black with white stroke) point to the photo-micrograph processing sequence; thin arrows point to the tissue/structure of interest. [file Image_2.JPEG]
